# Unbalanced inflammatory reaction could increase tissue destruction and worsen skin infectious diseases – a comparative study of leishmaniasis and sporotrichosis

**DOI:** 10.1038/s41598-018-21277-1

**Published:** 2018-02-13

**Authors:** F. N. Morgado, L. M. V. de Carvalho, J. Leite-Silva, A. J. Seba, M. I. F. Pimentel, A. Fagundes, M. F. Madeira, M. R. Lyra, M. M. Oliveira, A. O. Schubach, F. Conceição-Silva

**Affiliations:** 1 Laboratório de Imunoparasitologia, Instituto Oswaldo Cruz/FIOCRUZ, Rio de Janeiro, Brazil; 2 LaP Clin VigiLeish, Instituto Nacional de Infectologia Evandro Chagas/FIOCRUZ, Rio de Janeiro, Brazil; 3 Laboratório de Pesquisa em Leishmaniose, Instituto Oswaldo Cruz/FIOCRUZ, Rio de Janeiro, Brazil; 4 Laboratório de Micologia, Instituto Nacional de Infectologia Evandro Chagas/FIOCRUZ, Rio de Janeiro, Brazil

## Abstract

The clinical presentations of skin diseases produced by different pathogens, as American tegumentary leishmaniasis (ATL) and sporotrichosis can be similar and possibly influenced by the skin immune system (SIS). The aim of the study was to understand the underlying mechanisms of skin inflammation produced by different pathogens. We used immunohistochemistry to analyze 96 patients: a- localized cutaneous leishmaniasis (LCL-ATL); b- sporotrichoid cutaneous leishmaniasis (SCL-ATL); c-lymphocutaneous (LC-SP); d- fixed (F-SP) sporotrichosis. LCL-ATL and SCL-ATL had a significantly higher percentage of CD8, FasL and NOS2 than sporotrichosis. In contrast, LC-SP had a substantially higher percentage of CD4, BCl2 and neutrophils than ATL lesions. These results indicated some differences in the profile of the *in situ* immune response suggesting that SIS is a complex, adaptable system capable of different responses to intracellular or extracellular pathogens. However, regardless of the etiological agents, the inflammatory reaction and clinical manifestations can be similar. SCL-ATL and LC-SP presented similarities in both clinical presentation and *in situ* inflammatory profile (CD3, CD22, neutrophils, macrophages). The clinical presentation of ATL and sporotrichosis could be explained by a combination of factors both of the host SIS and the etiological agent. The unbalanced host parasite relationship could result in atypical manifestations of skin disease.

## Introduction

Immunologists have recently paid more attention to the importance of the skin for immune surveillance. In humans, the skin, which covers approximately 2 m^2^ and accounts for 16% of the body weight, is the largest organ of the body^1^. This organ has several immune systems, such as the skin immune system (SIS), skin-associated lymphoid tissue (SALT), and hair follicle immune system (HFIS)^1–6^. Consequently, the skin is now deemed essential for the development and selection of the immune response to several agents^7–14^.

As an immune surveillance organ, the skin continuously interacts with various infectious agents. Not surprisingly, some infectious and parasitic diseases primarily or secondarily target the skin^15–22^. For example, *Leishmania* spp. and *Sporothrix* spp cause two granulomatous skin diseases: American tegumentary leishmaniasis (ATL) and sporotrichosis (SP), respectively. Although these diseases share clinical similarities as ulcerated lesions that arise frequently in the limbs^23^, they differ in their duration and the number of lesions as well as the degree to which the skin is involved^20,23–25^. Since SP is caused by an extracellular fungus that occasionally enters phagocytic cells whereas ATL is caused by an obligatory intracellular parasite of mononuclear phagocytes, we hypothesized that these differences could elicit different SIS responses and, therefore, cause different clinical symptoms and signs. To test this hypothesis, we used immunohistochemistry to compare the *in situ* inflammatory reaction of active lesions in ATL and SP patients presenting a dissimilar clinical presentation in order to elucidate some aspects underlying the mechanisms of localized inflammation of the skin by different infectious agents.

## Results

### Sporotrichosis and American tegumentary leishmaniasis patients only partially differ in the aspect of lesions and duration of infection before diagnosis

**Figure 1:**
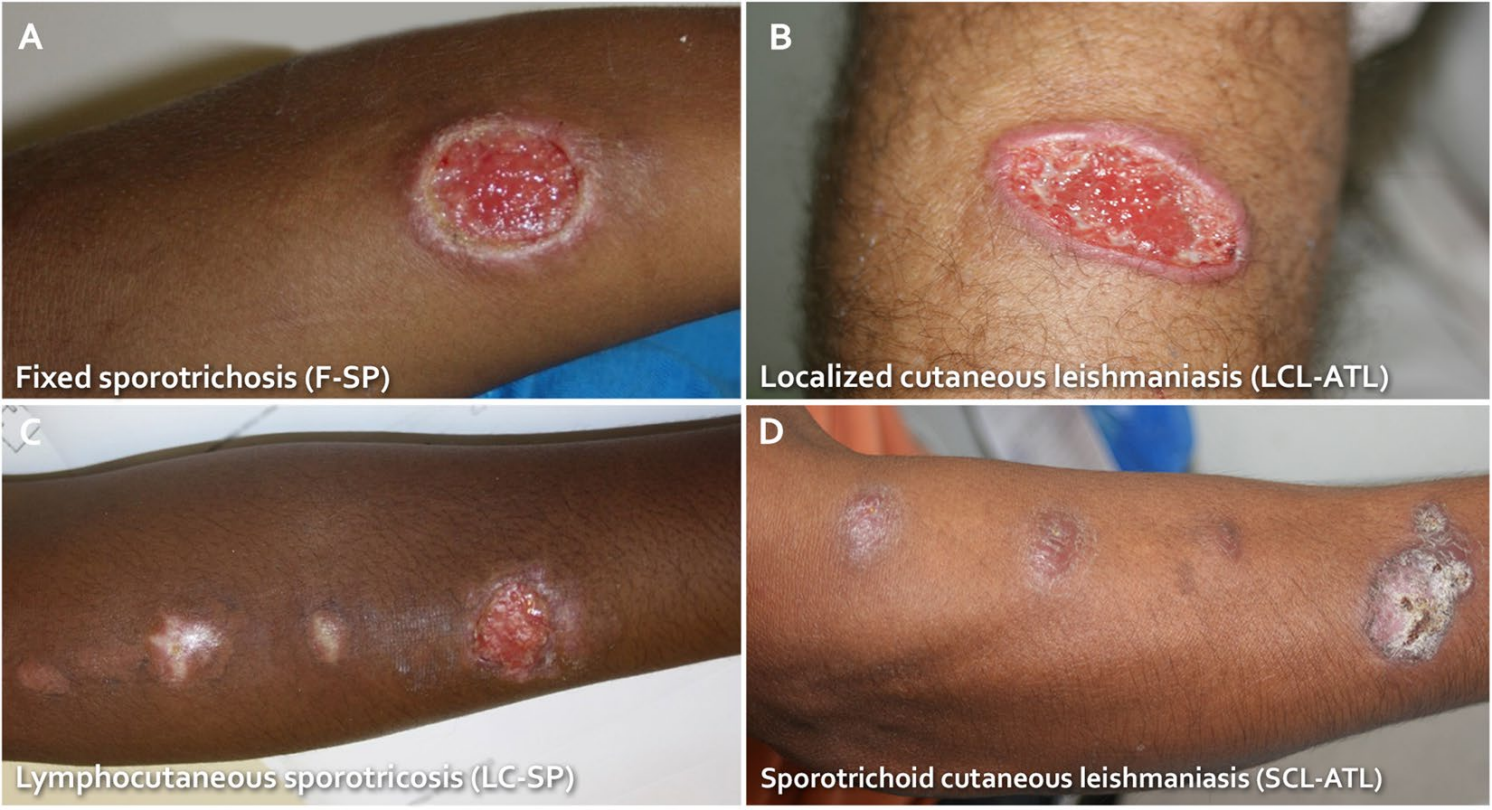
Comparison of the clinical presentations of patients with (**A**) a fixed form of sporotrichosis (F-SP), (**B**) localized cutaneous leishmaniasis (LCL-ATL), (**C**) lymphocutaneous (LC-SP) form of sporotrichosis and (**D**) sporotrichoid cutaneous leishmaniasis (SCL-ATL). Note the similarities between the different clinical forms of sporotrichosis and ATL.

LCL-ATL and F-SP commonly presented single and localized lesions without lymphatic involvement (Fig. 1A,B). SCL-ATL and LC-SP presented multiple lesions frequently associated with lymphangitis and more extensive lesions (Fig. 1C,D). All 4 groups of patients had similar age distributions (p > 0.05; Table 1). The duration of infection, time elapsed between the beginning of the cutaneous lesions and the attendance of the patient at the Instituto Nacional de Infectologia Evandro Chagas – (INI) and the diagnostic procedures, was different among the groups. Sporotrichosis patients showed more acute development since their evolution was shorter than that of the LCL-ATL patients (Table 1). However, SCL-ATL presented similar duration as LC-SP (p > 0.05). F-SP showed the shorter time of evolution (Table 1).

**Table 1:** Distribution of age and duration of infection in American tegumentary leishmaniasis and sporotrichosis patients.

Clinical data	LCL-ATL n = 30 Median (Range)	SCL-ATL n = 18 Median (Range)	F-SP n = 24 Median (Range)	LC-SP n = 24 Median (Range)	Kruskal-Wallis test
Age (Years)	39.5 (14–71)	40 (13–71)	29.5 (18–73)	40 (16–68)	p = 0.100
Duration of infection* (months)	2.0 (1–17)	2.0 (1–12)	1.1 (0.25–4.0)	1.0 (0.25–4.5)	**p = 0**.**002**

LCL-ATL: localized cutaneous leishmaniasis; SCL-ATL: sporotrichoid cutaneous leishmaniasis; F-SP: fixed sporotrichosis; LC-SP: Lymphocutaneous sporotrichosis.

*Elapsed Time between the beginning of the cutaneous lesions and the diagnostic procedures in INI-FIOCRUZ. Bonferroni post hoc test: F-SP vs. LCL-ATL: p = 0.045.

Mann-Whitney test LCL-ATL *vs*. LC-SP: p = 0.002; LCL-ATL *vs*. F-SP: p = 0.003.

### Sporotrichosis and American tegumentary leishmaniasis lesions can differ in the distribution of their inflammatory reactions

The inflammatory infiltrate in sporotrichosis lesions strongly predominated in the reticular dermis, near sweat glands and ducts, and around blood vessels, while that in ATL lesions it prevailed in the papillary dermis and was diffusely distributed throughout the lesion. However, since the inflammatory cells were heterogeneously distributed, there were no apparent differences in the cell density per mm^2^ of tissue in any of the four groups of patients with ATL or SP. Moreover, they were different from healthy skin that showed reduced cellularity organized as cell nests around cutaneous adnexa more concentrated in the papillary dermis (data not shown).

### The percentage composition of cell types and markers of *in situ* inflammatory reactions only partially differ when ATL and SP lesions are compared

**Figure 2:**
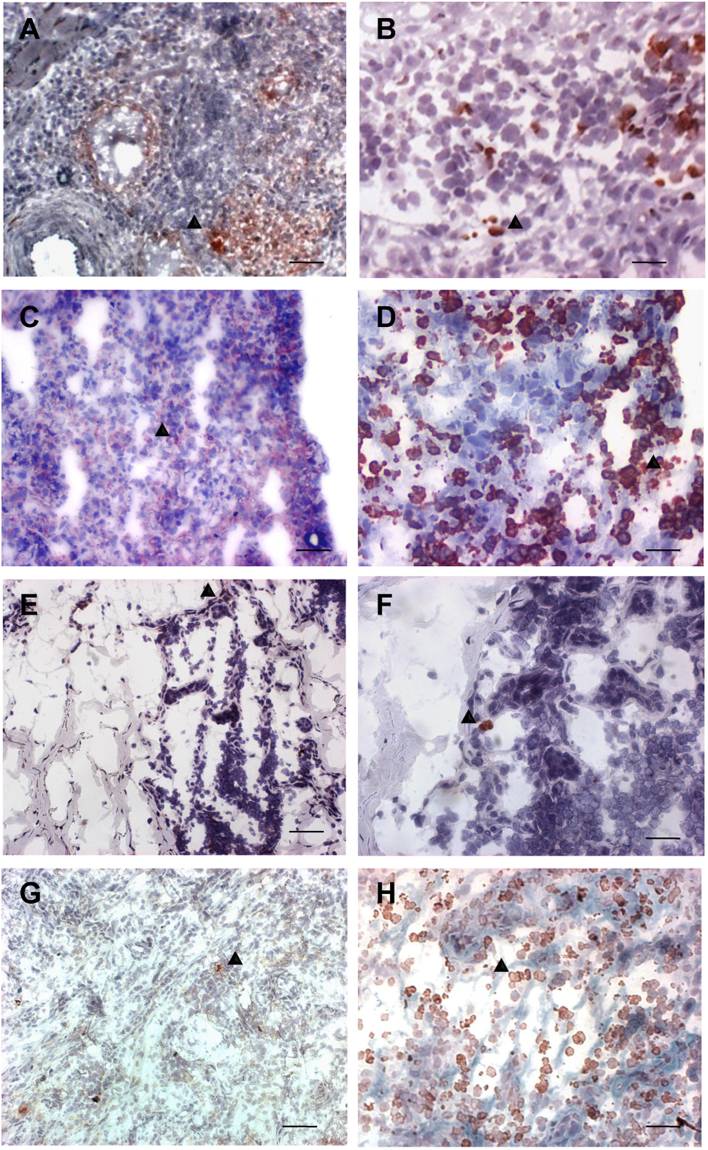
NOS2 expression (**A**,**C**,**E**,**G**) and neutrophils (**B**,**D**,**F**,**H**) in active lesions of LCL-ATL (**A**,**B**), SCL-ATL (**C**,**D**), F-SP (**E**,**F**) and LC-SP (**G**,**H**) patients. Magnification (**A**,**C**,**E**,**G**) 200× scale bar = 50 µm and (**B**,**D**,**F**,**H**) 400× scale bar = 25 µm. LCL-ATL: localized cutaneous leishmaniasis; SCL-ATL: sporotrichoid cutaneous leishmaniasis; F-SP: fixed sporotrichosis; LC-SP: Lymphocutaneous sporotrichosis. Arrows demonstrate examples of positive cells.

**Figure 3:**
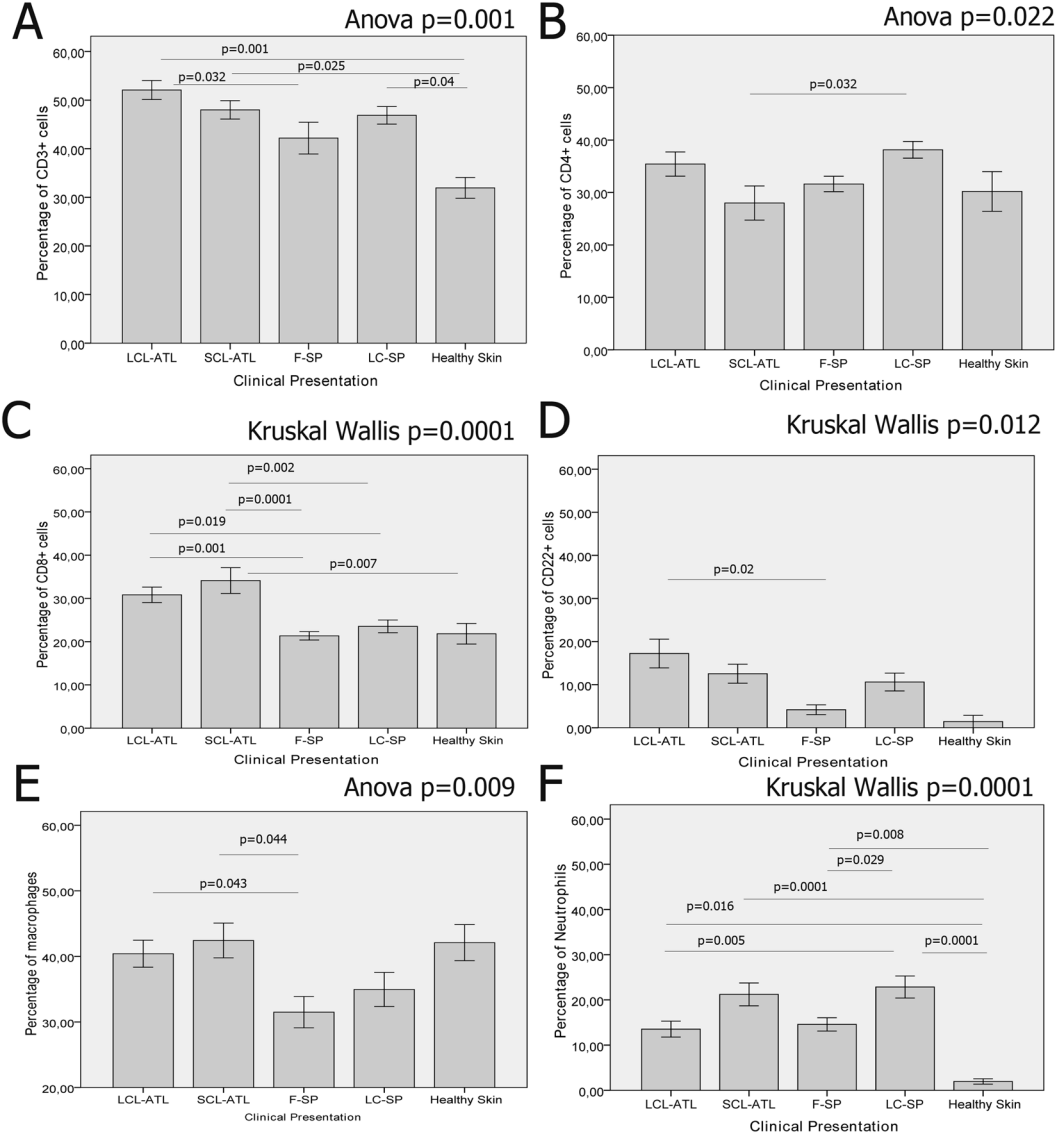
Percentage of (**A**) CD3^+^, (**B**) CD4^+^, (**C**) CD8^+^, (**D**) CD22^+^ cells, (**E**) macrophages (CD68^+^ cells) and (**F**) neutrophilic elastase-positive cells (neutrophils) Data represented as mean and SEM. P-value < 0.05 was considered statistically significant. LCL-ATL: localized cutaneous leishmaniasis; SCL-ATL: sporotrichoid cutaneous leishmaniasis; F-SP: fixed sporotrichosis; LC-SP: Lymphocutaneous sporotrichosis. Anova test was used for variables with normal distribution, otherwise Kruskal-Wallis test was applied. Bonferroni test was used as post hoc test. Pis modi aliquatem ad magnate erupis nulpari busam, nimus aut aut odi comniatur sita.

**Figure 4:**
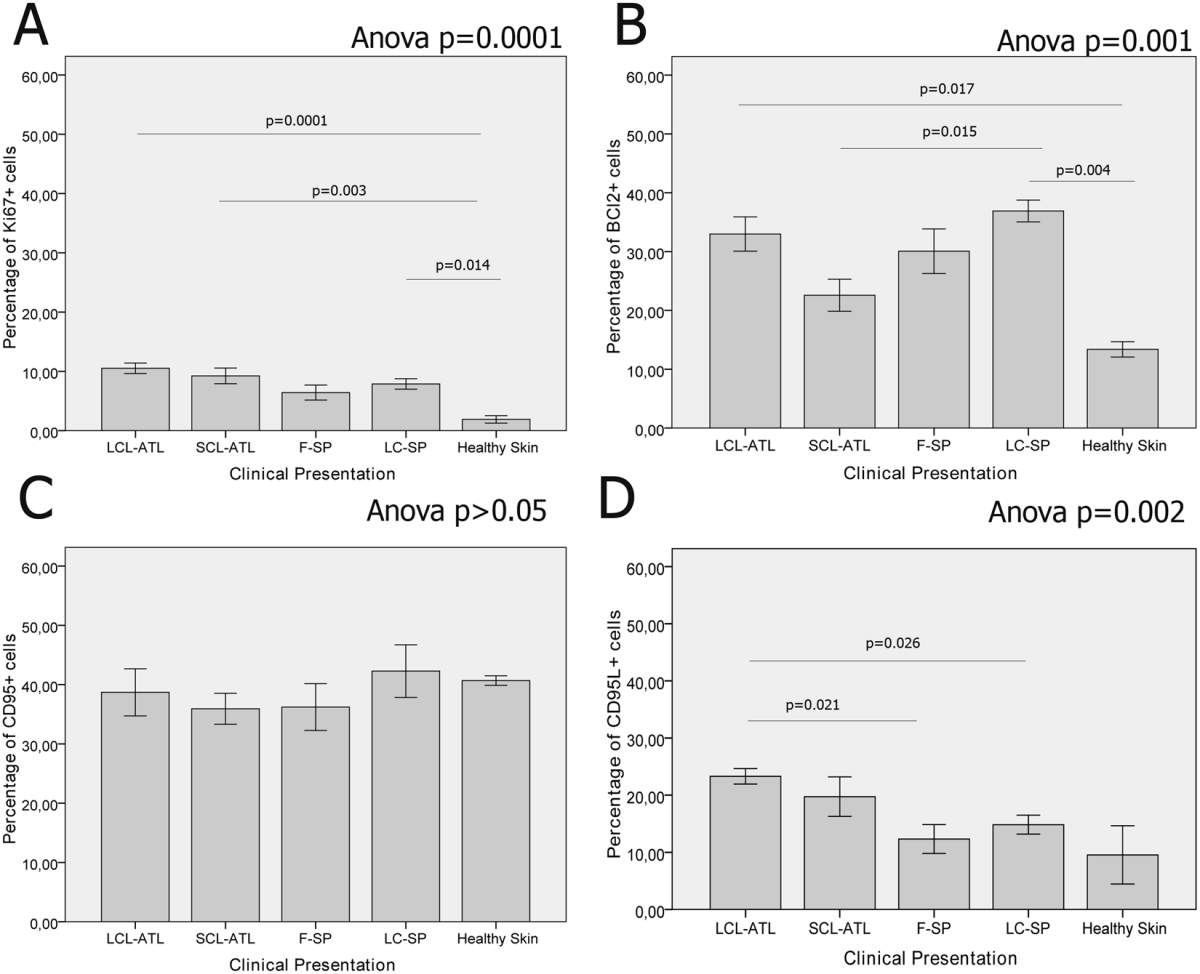
Percentage of (**A**) Ki67^+^, (**B**) BCl2^+^, (**C**) CD95^+^ (Fas^+^ cells), (**D**) CD95L^+^ (FasL^+^ cells). Data represented as mean and SEM. P-value < 0.05 was considered statistically significant. LCL-ATL: localized cutaneous leishmaniasis; SCL-ATL: sporotrichoid cutaneous leishmaniasis; F-SP: fixed sporotrichosis; LC-SP: Lymphocutaneous sporotrichosis. Anova test was used for variables with normal distribution, otherw ise Kruskal-Wallis test was applied. Bonferroni test was used as post hoc test.

We observed significant differences as well as similarities in the percentage composition of cell types and markers between LCL-ATL, SCL-ATL, F-SP and LC-SP lesions (Tables 2–3 and Suplemmentary Table S1) (Figs 2–4). All four patient groups presented a higher percentage of CD3^+^ cells than healthy skin (p < 0.05). LCL-ATL patients had the highest percentage of CD3^+^ cells and it was significantly different from F-SP patients (p = 0.012; Mann-Whitney test) (Fig. 3A and Table 2). Finally, CD3^+^ cells were similar in SCL-ATL and LC-SP.

**Table 2:** Cell types and inflammatory markers in American tegumentary leishmaniasis and sporotrichosis lesions. Data shown as median and range.

Cell types or inflammatory markers	LCL-ATL N = 18	SCL-ATL N = 18	F-SP N = 24	LC-SP N = 24	Healthy N = 9
CD3 % and range	51.8 31.7–70.0	46.8 39.3–65.5	41.9 27.5–65.8	47.6 27.5–62.1	32.8 26.1–36.2
CD4 % and range	35.0 16.3–59.9	24.7 8.9–50.5	29.8 17.8–44.6	39.6 22.3–54.4	24.1 19.7–50.5
CD8 % and range	32.14 15.3–48–5	39.2 14.0–49.3	19.4 15.6–31.2	21.8 12.0–42.9	21.1 13.2–32.4
CD22 mm^−2^ and range	11.2 0–46.0	13.1 1.8–30.0	2.9 0–9.4	7.5 1.0–25.0	0 0–4.3
Macrophages % and range	45.2 14.2–53.8	43.7 29.0–57.2	30.2 9.3–62.2	36.0 16.3–64.7	43.2 28.8–54.7
Neutrophils % and range	12.2 0.9–37.7	17.7 9.9–43.2	16.9 1.9–26.5	21.5 4.3–49.6	1.3 0–5.2
CD1a mm^−2^ and range	8.5 0.5–56.0	15.5 3.0–38.0	4.0 0–8.0	2.0 0–11.0	ND
Bcl-2% and range	35.9 6.7–68.4	25.0 2.3–40.9	28.6 12.0–48.0	37.4 22.6–48.0	13.9 10.1–16.3
Ki-67 % and range	9.7 4.9–24.0	8.3 3.2–19.9	5.7 1.8–15.5	7.3 1.7–16.9	2.1 0–4.2
CD95 % and range	39.7 1.4–78.4	36.9 15.1–50.5	39.0 17.2–50.4	40.4 9.5–75.0	40.1 39.5–43.0
CD95L % and range	23.7 9.9–36.2	24.8 1.8–36.6	10.7 3.4–25.5	13.6 4.2–29.0	7.2 0–23.9

LCL-ATL^a^: localized cutaneous leishmaniasis; SCL-ATL^b^: sporotrichoid cutaneous leishmaniasis; F-SP^c^: fixed sporotrichosis; LC-SP^d^: Lymphocutaneous sporotricosis.

**Table 3:** Intensity of NOS2 expression in American tegumentary leishmaniasis and sporotrichosis lesions. Data shown as number of cases.

Clinical presentation	Intensity of NOS2 expression				
	Negative	Discrete	Moderate	Intense	Very intense
LCL-ATL	0	6 (20%)	9 (30%)	11 (36.7%)	4 (13.3%)
SCL-ATL	0	0	3 (20%)	6 (40%)	6 (40%)
F-SP	3 (27.2%)	4 (36.4%)	4 (36.4%)	0	0
LC-SP	1 (5.6%)	3 (16.7%)	8 (44.4%)	6 (33.3%)	0
Healthy	0	6 (75%)	2 (25%)	0	0

LCL-ATL: localized cutaneous leishmaniasis; SCL-ATL: sporotrichoid cutaneous leishmaniasis; F-SP: fixed sporotrichosis; LC-SP: Lymphocutaneous sporotrichosis

Chi-square p = 0.0001; Pearson Chi-square: 79.131.

For six patients from LC-SP, thirteen patients from F-SP and one patient from healthy group, NOS2 expression was not performed.

The highest percentage of CD4^+^ cells was found in LC-SP lesions (Fig. 3B and Table 2). CD4^+^ cells were similar in LCL-ATL and F-SP. SCL-ATL lesions showed the lowest percentage of CD4^+^ cells in lesions of ATL or SP patients, similar to that of healthy skin. On the other hand, SCL-ATL had more CD8^+^ than CD4^+^ cells, and more CD8^+^ cells than the other groups (Fig. 3C and Table 2). In general, the percentage of CD8^+^ cells was higher in the leishmaniasis than in the sporotrichosis lesions. Moreover, the number of CD8^+^ cells in the sporotrichosis lesions was similar to that of the healthy skin.

B lymphocytes concentration was more intense in LCL-ATL lesions (Fig. 3D and Table 2). It was similar in SCL-ATL and LC-SP, and higher than that in F-SP and in healthy skin. These last two were comparable regarding B lymphocytes concentration.

Excepting F-SP, there were no differences in the percentage of macrophages among the groups including healthy skin (Fig. 3E and Table 2). However, it is noteworthy emphasizing that cellularity per tissue area in healthy skin was markedly reduced when compared to the lesion groups, producing a difference when the absolute number of cells were counted. On the other hand, the percentage of neutrophils was higher in SCL-ATL and LC-SP than in the other groups (Fig. 2B,D,F,H and F and Table 2). It was lower and similar in LCL-ATL and F-SP groups. Finally, all lesions groups showed a higher percentage of neutrophils than healthy skin.

NOS2 expression was higher in leishmaniasis than in sporotrichosis lesions (Table 3 and Fig. 2A,C,E,G). The highest expression was found in SCL-ATL lesions and 80% were classified as intense or very intense expression, followed by LCL-ATL (50%) and LC-SP (33.3%). F-SP lesions showed a similar expression of NOS2 when compared to healthy skin.0

All lesion groups showed more BCl-2^+^ and Ki67^+^ cells than healthy skin (Fig. 4A,B and Table 2). BCl-2^+^ cells were more common in LC-SP and less frequent in SCL-ATL. Although Fas expression was similar among the groups (Fig. 4C), FasL was more expressed in leishmaniasis lesions than sporotrichosis or healthy skin (Fig. 4D). There were no differences between sporotrichosis lesions and healthy skin, regarding FasL.

## Discussion

In this study, we compared the *in situ* inflammatory response of ATL and SP, cutaneous lesions in order to evaluate how the skin immune system reacts to pathogens with different natures and in patients with diverse or similar clinical aspects. Our results have pointed to a general and similar inflammatory skin reaction with some differences according to the infection/clinical presentation. Although both infections primarily target the skin, they can present different degrees of *in situ* granulomatous reactions and different courses of disease. SP is often a subacute infection, which is characterized by an exudative reaction, whereas ATL is a chronic disease, which is characterized by a long duration of infection and the presence of non-exudative ulcers^26–28^. Furthermore, their infectious agents mainly affect different cellular compartments (extracellular and intracellular). As a result, we hypothesized that differences in the cellular composition and markers of the *in situ* inflammatory activity in ATL and SP lesions could reveal two different patterns: intracellular and extracellular responses. However, our results pointed that sometimes their arrangements can be similar as observed for SCL-ATL and LC-SP. In the present study, even pathogens with different natures could elicit similar immune responses and clinical aspects of lesions, which could reflect a host background. Furthermore, the same pathogen can also elicit different degree of immune response and severity of clinical lesions. Although the potential of clones/isolates from the same pathogens in eliciting different degrees of tissue damage should be considered^29–31^, it is now well established that the clinical presentation is a consequence of host-parasite balance^32–34^. In this context, it has been recently demonstrated^35^ the role of Th17 cells in pathogenesis and fungal burden control in sporotrichosis.

We also observed differences in the clinical presentation of ATL and SP. For example, ATL lesions developed more slowly than the SP lesions. In fact, after only 2 weeks of infection, 6 SP patients had already developed lesions. On the other hand, in ATL lesions, the infection usually occurred more than 2 months before the patient’s first medical visit. These observations suggest a relationship between the nature of the etiological agent and the immune response leading to the development of acute, subacute, or chronic lesions.

Our results showed that ATL lesions elicited a pattern of inflammatory response that is characterized by CD3^+^, CD8^+^, and FasL^+^ cells as well as higher NOS2 expression than SP lesions. As *Leishmania* spp. infects and thrives inside phagocytes, these cells should be activated or eliminated to reduce the parasite burden^9,24,36^. Since the cellular immune response and cytotoxicity have major roles in the effector immune response to control intracellular pathogens^37^, this profile would characterize the intracellular pattern of the skin immune response against intracellular pathogens. However, excess of NOS2 expression as observed in SCL-ATL and LC-SP when compared to counterpart’ lesions (LCL-ATL and F-SP) could be associated to more extensive and severe lesions suggesting an unbalanced/uncontrolled immune response. As macrophages were similar in all studied groups, we can suggest that these cells might be distinctly activated. In this context, M1 and M2 macrophages have already been demonstrated in literature^38–41^, but other analyses should be done to better clarify this hypothesis.

On the other hand, our results demonstrated that LC-SP lesions predominantly elicited an pattern of response that is characterized by more neutrophils and CD4^+^ cells, and fewer markers of cytotoxic response and NOS2 expression than ATL lesions. In response to extracellular pathogens like helminthes, Gram-negative bacteria, some fungi or some protozoa, it is thought that secretory factors, antibodies, enzymes, antibiotic peptides, and various cells, such as mast cells, neutrophils, and eosinophils, are responsible for reducing the microorganism burden^42^. However, there are few studies on the immune response in sporotrichosis. In a murine model of sporotrichosis infection, thymus-derived cells are involved in resistance to this infection, which suggests that cellular immunity plays an important role in host resistance to this pathogen^43–45^. Interestingly, SCL-ATL showed similar quantities of neutrophil elastase expression to LC-SP, and in both they were more intense than in the other groups. Neutrophil elastase can cause tissue liquefaction (pus)^25^. Although tissue damage is usually harmful, small amounts of neutrophil-mediated tissue damage are beneficial because they destabilize collagen fibers leading to the collapse of capillaries and lymphatic vessels and confine the infectious agent to a local, toxic environment where can be destroyed^46^. In addition, neutrophils also secrete chemotactic factors for T cells and can induce T cell activation via interferon γ^47^. The cooperation of macrophages and neutrophils in order to eliminate parasites or fungal cells respectively in leishmaniasis or sporotrichosis, has also been suggested^25,48–52^. However, our results showed that the highest concentration of neutrophils occurred in LC-SP and SCL-ATL lesions, which are both characterized by a regional lymphatic spread. It is worth note that neutrophil enzymatic contents are able to degrade the extracellular matrix, leading to an easier migration of immune cells^53^. However, this function also could facilitate fungus spread, through lymphatic vessels^54,55^. As a result, even in the presence of the correct stimulus, an unbalanced inflammatory reaction could increase tissue destruction and facilitate fungal or parasite spread.

Interestingly, despite the low number of B-lymphocytes their concentration was higher in LCL-ATL lesions, followed by SCL-ATL and LC-SP both in a similar degree. B lymphocytes and their secreted antibodies could play a role in parasite burden control in leishmaniasis opsonizing occasional extracellular amastigotes, inducing phagocytosis, complement activation or NK cells stimulation and interaction with T cells^56–59^. In sporotrichosis, the role of B cells and imunoglobulins were verified. The expression of specific immunoglobulins increases as the fungal load decreases in mice, which suggests that the humoral immune response could be related to pathogen elimination or to secondary protective mechanisms^60^.

Collectively, our results suggested that the SIS is a complex, adaptable structure that is capable of optimizing the response to intracellular or extracellular pathogens. However, an unbalanced inflammatory reaction could increase tissue destruction and worsen the disease.

Although these response models can account for the classical clinical presentation of LCL-ATL and LC-SP, some patients have atypical forms^25,43–45^ with different degrees of inflammation when compared with the typical clinical presentation. In this regard, F-SP lesions have an intermediate severity and development time when compared to LC-SP and ATL lesions. Consistent with these characteristics, the *in situ* inflammatory reaction in F-SP lesions exhibited fewer macrophages, CD3^+^, CD8^+^, and FasL^+^ cells as well as lower NOS2 expression than ATL lesions and fewer neutrophils and B cells than LC-SP lesions. In addition, our previous study^24^ showed that F-SP lesions have lower fungal burden than LC-SP lesions as well as clinical similarities (localized lesions without lymphatic alterations) with LCL-ATL lesions. On the other hand, when compared to F-SP, LC-SP lesions presented higher fungal burden and are clinically characterized by numerous skin nodules or plaques, following regional lymphatic dissemination, with an elevated degree of necrosis. On the basis of these findings, we hypothesize that the SIS response in patients with the F-SP rapidly controls the fungal burden, which reduces the severity of lesions and the exudative reaction, thereby promoting a more balanced immune response. Although this model of the immune response might not be completely applicable to the sporotrichoid form of ATL, it should be noted that even in SCL-ATL, the lesions are less exudative and develop more chronically than LC-SP lesions.

We cannot eliminate the possibility that different pathogen isolates could affect the SIS response to infection. However, at Rio de Janeiro State, *Leishmania braziliensis* is the causative agent of almost all cases and all isolates from ATL lesions included in our study were characterized as *Leishmania braziliensis*. Although the suggestion that different human *Sporothrix* spp isolates can affect both, the immune response and the development of infection in experimental sporotrichosis^29^, there is no clear relationship between human isolates and clinical presentation when genotype, protein expression, or antifungal susceptibility patterns are considered^33,61^. Moreover, in epidemic of sporotrichosis in Rio de Janeiro previous studies identified *S*. *brasiliensis* as most frequent species (up to 93.5% of the cases) by molecular analyses^62,63^. These results corroborate with data from Marimon and coworkers (2007) in a study that described the new species of complex *Sporothrix* being of 127 *Sporothrix* strains collected from several parts of the world reported only *S*. *brasiliensis* among the tested isolates from Rio de Janeiro^64^.

In addition, we cannot exclude the possibility that, during infection, *Leishmania* amastigotes and *Sporothrix* spp yeasts can be transiently localized in the extracellular and intracellular milieu, respectively. This possibility could be important to control the parasite and fungal burden. However, the duration of the transience and the effects on the skin immune response are not known. Though, our results suggested that the clinical presentation of infectious skin diseases could be due to a combination of factors from both, the host SIS and the etiological agent.

Therefore, we could draw two conclusions. First, the SIS is a complex, adaptable structure that is capable of responding with plasticity to intracellular or extracellular pathogens in order to control microorganism burden. Second, the clinical presentation of infectious skin diseases could result from a combination of factors from both, the host SIS and the etiological agent. Since more severe lesions from both SP and ATL presented an important concentration of neutrophils, or CD8^+^ T cells and NOS2 expression, respectively, our results also suggest that, unbalanced host SIS - parasite relationship can lead to more severe manifestations of skin infectious diseases.

## Material and Methods

### Patients

Patients were diagnosed and followed up in the outpatient clinic of the Laboratório de Pesquisa Clinica e Vigilância em Leishmanioses (LaP Clin VigiLeish), Instituto Nacional de Infectologia Evandro Chagas (INI), Fundação Oswaldo Cruz (FIOCRUZ), Rio de Janeiro, Brazil. A total of 105 individuals were divided into 5 groups: (1) Localized cutaneous leishmaniasis (LCL-ATL n = 30); (2) Sporotrichoid cutaneous leishmaniasis (SCL-ATL n = 18); (3) Fixed cutaneous sporotrichosis (F-SP n = 24); (4) Lymphocutaneous sporotrichosis (LC-SP n = 24); (5) Healthy skin (n = 9) from esthetical surgery. The study was approved by the Ethics Committee of INI-FIOCRUZ (04/2001) and all patients provided informed written consent. All methods were performed in accordance with the relevant guidelines and regulations related to researches using humans.

### Tissue samples

Samples were obtained from the primary lesion from patients at the time of their investigative procedures for diagnosis. Normal skin was obtained from healthy individuals during esthetical surgery procedures. These samples were prepared for the following analyses: (1) histopathology (stored in 10% formalin buffer), (2) immunohistochemistry (stored at −196 °C in OCT medium (Tissue-Tek, Sakura Finetek, Torrance, CA, USA), and (3) collected in sterile saline and cultured to isolate fungal cells and *Leishmania* spp. In order to isolate fungal cells, the samples were cultured at 28 °C in Sabouraud Dextrose Agar supplemented with 20 U·mL^−1^ penicillin and 40 µg·mL^−1^ streptomycin (Sigma, St. Louis, MI, USA) during the hyphal phase. Then followed by growth at 37 °C in Brain Heart Infusion medium (Sigma) until the yeast like phase. The fungal strains obtained of six patients used in this study were characterized at species level according to Oliveira and collaborators^62^ using polifasic taxonomy. Briefly, fungi were sub-cultured on Potato dextrose agar and Mycobiotic agar (both from DifcoTM BD/Sparks MD, USA), and then identified by phenotypic and genotypic characteristics (macro and micromorphology, thermotolerance, carbohydrate assimilation and molecular assay). All of them were characterized as S. brasiliensis**”**To isolate *Leishmania* spp., the samples were cultured in biphasic medium Novy-MacNeal-Nicolle (NNN)/Schneider’s insect medium (Sigma) at 28 °C. Twenty-five isolates were typing through *multilocus* enzyme electrophoresis as *L*. *braziliensis*.

### Histopathology

Formalin-fixed samples were stained with hematoxylin-eosin, and examined with a light microscope (Carl Zeiss Axioskop, Jena, Germany).

### Immunohistochemistry

Immunohistochemistry was performed as described previously^24^. Three-micrometer-thick sections were mounted on silanized slides (Dakocytomation, Carpinteria, CA, USA), fixed in acetone, and hydrated in phosphate buffered saline (PBS, pH 7.4). After blocking endogenous peroxidase with peroxidase blocking reagent (Dakocytomation) and nonspecific staining with normal goat serum (Zymed, San Francisco, CA, USA), the slides were incubated with the following antibodies: CD3^+^ (clone UCHT1), CD4^+^ (clone MT310) and CD8^+^ (clone DK25) T lymphocytes, CD22^+^ (clone 4KB128) B lymphocytes, CD68^+^ (clone KP1) macrophages, Bcl-2^+^ (clone 124), Ki67^+^ (clone Ki-S5) and neutrophil elastase (clone NP57 - neutrophils) (Dakocytomation); nitric oxide synthase 2 (NOS2) (clone 6) (BD Transduction Laboratories, KY, USA); Fas (clone DX2), and FasL (clone G247–4) (BD Biosciences Pharmingen, San Jose, CA, USA). After that, the specimens were incubated with a biotinylated secondary antibody (goat anti-mouse IgG or goat anti-rabbit IgG – both from Zymed) followed by a streptavidin-biotin-peroxidase complex (ABC kit, Dakocytomation) and aminoethylcarbazole (AEC kit, Zymed). Subsequently, the slides were counterstained with Mayer’s hematoxylin (Dako) and examined under a light microscope (Zeiss). The percentage of stained cells was determined among 500 mononuclear cells. The intensity of NOS2 staining was measured in 10 microscope fields (200× magnification) and scored as discrete (1 positive), moderate (2 or 3 positive areas), intense (4 or 5 positive areas), or very intense (>5 positive areas), as described previously^24^. All experiments were replicated at least twice and the suppression of the primary antibody served as a negative control.

### Statistical analysis

Statistical analyses were calculated with SPSS24 for Windows (SPSS, Inc., Chicago, IL, USA). Kolmogorov Smirnov test was used to evaluate the distribution of variables. The Mann-Whitney or t-Student tests and Kruskal Wallis or Anova tests and Bonferroni post hoc test were used to compare the groups. Data are reported as median, SEM and range. The p-value cutoff for statistical significance was 0.05.

## Electronic supplementary material

Supplementary Table S1
