# Supplementary material for: Unbalanced inflammatory reaction could increase tissue destruction and worsen skin infectious diseases – a comparative study of leishmaniasis and sporotrichosis
Source: Sci Rep. 2018 Feb 13;8:2898. doi: 10.1038/s41598-018-21277-1 (PMC5811542; doi:10.1038/s41598-018-21277-1)
Supplement: Supplementary file 1 — Supplementary Table S1 [file 41598_2018_21277_MOESM1_ESM.pdf]

Unbalanced inflammatory reaction could increase tissue destruction and worsen skin infectious diseases – a comparative study of leishmaniasis and sporotrichosis

F.N. Morgado[1,3,#]; L.M.V. de Carvalho[1,#], J. Leite-Silva[1]; A.J. Seba[1]; M.I.F. Pimentel[2]; A. Fagundes[2]; M.F. Madeira[2]; M.R. Lyra[2]; M.M. Oliveira[4]; A.O. Schubach[2]; F. Conceição-Silva[1]*

Supplementary Material

Supplementary Table S1. P-values of two-independent group comparisons by Mann-Whitney or T-student tests.

Marker	LCL-ATL *vs* SCL-ATL	LCL-ATL *vs* F-SP	LCL-ATL *vs* LC-SP	LCL-ATL *vs* Healthy	SCL-ATL *vs* F-SP	SCL-ATL *vs* LC-SP	SCL-ATL *vs* Healthy	F-SP *vs* LC-SP	F-SP *vs* Healthy	LC-SP *vs* Healthy	Test
CD3	> 0.05	**0.012**	> 0.05	**0.001**	> 0.05	> 0.05	**0.001**	> 0.05	> 0.05	**0.002**	T-student
CD4	> 0.05	> 0.05	> 0.05	>0.05	> 0.05	**0.011**	> 0.05	**0.004**	> 0.05	**0.029**	T-student
CD8	> 0.05	**0.0001**	**0.006**	**0.012**	**0.001**	**0.004**	**0.015**	> 0.05	> 0.05	> 0.05	Mann-Whitney
CD22	> 0.05	**0.016**	> 0.05	**0.035**	**0.008**	> 0.05	**0.017**	**0.048**	> 0.05	**0.047**	Mann-Whitney
MO	> 0.05	**0.006**	> 0.05	> 0.05	**0.006**	> 0.05	> 0.05	> 0.05	**0.019**	> 0.05	T-student
NEU	**0.019**	> 0.05	**0.002**	**0.0001**	> 0.05	> 0.05	**0.0001**	**0.014**	**0.001**	**0.0001**	Mann-Whitney
CD1a	**0.011**	> 0.05	**0.002**	> 0.05	**0.0001**	**0.0001**	> 0.05	> 0.05	> 0.05	> 0.05	T-student test
Bcl-2	**0.028**	> 0.05	> 0.05	**0.0001**	> 0.05	**0.0001**	**0.007**	> 0.05	**0.002**	**0.0001**	T-student
Ki-67	> 0.05	**0.016**	**0.049**	**0.0001**	> 0.05	> 0.05	**0.0001**	> 0.05	**0.006**	**0.0001**	T-student
CD95	> 0.05	> 0.05	> 0.05	> 0.05	> 0.05	> 0.05	> 0.05	> 0.05	> 0.05	> 0.05	T-student
CD95L	> 0.05	**0.001**	**0.0001**	**0.002**	> 0.05	> 0.05	> 0.05	> 0.05	> 0.05	> 0.05	T-student

LCL-ATL: localized cutaneous leishmaniasis; SCL-ATL: sporotrichoid cutaneous leishmaniasis; F-SP: fixed sporotrichosis; LC-SP: Lymphocutaneous sporotricosis
